# Comparative Outcomes of Budesonide MMX versus Prednisolone for Ulcerative Colitis: Results from a British Retrospective Multi-Centre Real-World Study

**DOI:** 10.3390/jcm10194329

**Published:** 2021-09-23

**Authors:** Konstantina Rosiou, Elaine Ong Ming San, Aditi Kumar, Kim Esquivel, Saima Almas, Daniel Stokes, Tze Ng, Nishani Jayasooriya, Ian Ranasinghe, Richard Pollok, Matthew Brookes, Christian P. Selinger

**Affiliations:** 1 Leeds Gastroenterology Institute, Leeds Teaching Hospitals, Leeds LS9 7TF, UK; konstantina.rosiou@nhs.net (K.R.); e.ongmingsan@nhs.net (E.O.M.S.); 2 Department of Gastroenterology, Royal Wolverhampton Hospital, Wolverhampton WV10 0QP, UK; aditikumar@nhs.net (A.K.); kim.esquivel@nhs.net (K.E.); s.almas1@nhs.net (S.A.); dstokes2@nhs.net (D.S.); matthew.brookes@nhs.net (M.B.); 3 Department of Gastroenterology, St Georges Hospital, London SW17 0QT, UK; tze.ng@stgeorges.nhs.uk (T.N.); nishani.jayasooriya@nhs.net (N.J.); ian.ranasinghe@stgeorges.nhs.uk (I.R.); richard.pollok@nhs.net (R.P.); 4 Research Institute in Healthcare Science (RIHS), University of Wolverhampton, Wolverhampton WV1 1LY, UK

**Keywords:** ulcerative colitis, inflammatory bowel disease, prednisolone, Budesonide MMX, Cortiment, COVID-19

## Abstract

During the COVID-19 pandemic many IBD units chose Budesonide MMX (Cortiment) as the first-line treatment for flares of ulcerative colitis (UC) in outpatients for its favourable side effect profile. This retrospective study of all UC patients treated with oral steroids between 1 March 2019–30 June 2019 and 1 March 2020–30 June 2020 aimed to compare Cortiment with Prednisolone in routine clinical practice. Outcomes included the need for hospitalisation for acute severe ulcerative colitis, symptoms at four weeks and end of treatment, and the need for rescue Prednisolone. The 2019 and 2020 cohorts did not differ at the baseline. Cortiment prescriptions rose from 24.5% in 2019 to 70.1% in 2020 (*p* < 0.001). At week four there were significant differences between 2019 and 2020 in mean bowel frequency (3.49 vs. 5.85, *p* = 0.001), rectal bleeding <50% (89.7% vs. 73.1% of patients, *p* = 0.039), and physician global assessment (PGA) (39.2% vs. 19.8% in remission, *p* = 0.045). There was no significant difference in hospital admissions, rectal bleeding, and PGA at week eight. Rescue Prednisolone was required in 10% of Cortiment patients in 2019 vs. 31.3% in 2020 (*p* = 0.058). Active IBD is associated with worse COVID-19 outcomes prompting the careful evaluation of the choice of first-line steroid for UC, as Cortiment was associated with worse outcomes at four weeks.

## 1. Introduction

Ulcerative colitis (UC) is a chronic inflammatory bowel disease (IBD) that causes mucosal inflammation in the colon, starting distally in the rectum with a continuous extension more proximally, and typically comes with periods of remission and relapse [1,2]. Since the Truelove and Witts pivotal study first demonstrated their efficacy in patients with UC [3], oral corticosteroids have been used for the induction of remission in UC and are currently recommended in patients with mild to moderate UC in whom induction with 5-aminosalicylic acid (5-ASA) failed and in patients with moderate to severe UC [1,2]. However, conventional corticosteroids such as Prednisolone have significant side effects that limit their use. These include, but are not limited to, fluid retention, mood and sleep disturbance, acne, glucose intolerance, dyspepsia, osteoporosis, and susceptibility to infections [4].

Budesonide MMX (Cortiment) is an oral formulation of Budesonide that uses a multi-matrix system (MMX) technology in order to extend the release of Budesonide throughout the colon. The CORE I and CORE II trials demonstrated that Cortiment was safer and more effective than placeboes in inducing clinical and endoscopic remission in patients with active mild to moderate UC [5,6]. Current guidelines suggest that Cortiment can be considered as an alternative to conventional steroids in patients with mild to moderate disease who are intolerant to, or fail to respond to, aminosalicylates [1,2]. Cortiment is an appealing therapeutic option since it has a lower rate of systemic adverse effects compared to conventional steroids and is not associated with appreciable adrenal suppression [2,7]. However, there are currently no adequately powered head-to-head trials comparing Cortiment to conventional corticosteroids.

The first outbreak of the COVID-19 pandemic, caused by the severe acute respiratory syndrome coronavirus 2 (SARS-CoV-2), resulted in many changes in the conventional management of patients with UC, with a trend to avoiding endoscopy and reliance on clinical symptom assessments. [8,9,10,11]. Results from the Surveillance Epidemiology of Coronavirus Under Research Exclusion for Inflammatory Bowel Disease (SECURE-IBD) registry demonstrated that previous use of systemic corticosteroids is a risk factor for adverse COVID-19 outcomes [12]. Guidance from the British Society of Gastroenterology (BSG) for the management of IBD during the COVID-19 pandemic suggested avoiding systemic corticosteroids if possible and considering Cortiment as an alternative in UC [8].

The aim of this retrospective multi-centre British study was to assess the changes in steroid prescribing patterns during the COVID-19 pandemic and compare outcomes between Budesonide MMX (Cortiment) versus Prednisolone for the treatment of UC in a ‘real world’ setting.

## 2. Materials and Methods

We included patients from the IBD units of three representative UK hospitals (Leeds Teaching Hospitals, Royal Wolverhampton Hospitals, and St George’s Hospital, London). Hospital-based prescriptions for Cortiment and Prednisolone were extracted from computerised outpatient hospital pharmacy records for the time periods from 1 March 2019–30 June 2019 and 1 March 2020–30 June 2020. All adult patients treated with oral steroids (Prednisolone or Cortiment) for a flare in UC were included in the study. Patients treated with oral steroids for all other reasons apart from UC, i.e., Crohn’s disease, autoimmune pancreatitis, etc., were excluded.

Baseline data collected at the time of steroid prescription included: age, sex, phenotype (E1/E2/E3 according to Montreal classification), IBD medications (5-ASA, Thiopurines, Infliximab, Adalimumab, Golimumab, Vedolizumab, Tofacitinib, Ustekinumab) including use at the time of steroid prescription and previous use, medication changes within the last four weeks prior to steroid prescription, baseline symptoms (partial Mayo score including bowel frequency, rectal bleeding, and physician global assessment), steroid prescribed (Cortiment/Prednisolone), intended Prednisolone tapering regimen, and changes to medication at the time of steroid prescription (5-ASA dose alteration, rectal therapy alteration, commencement of biologic therapy or increase in biologic dose).

Follow up data included: the need for hospital admission for acute severe ulcerative colitis, symptom improvement within four weeks, symptoms at four weeks (partial Mayo score if available), need for rescue Prednisolone at four weeks (Cortiment group only), flare after initial improvement, symptoms at end of 6–8 weeks treatment episode (rectal bleeding and physician global assessment if available), need for rescue Prednisolone at eight weeks (Cortiment group only), and documented steroid side-effects.

### 2.1. Outcomes

The primary outcome was symptomatic improvement at four weeks of therapy without the need for rescue therapy in Cortiment-treated patients.

Secondary outcomes included:

- The proportion of Cortiment prescription in 2019 versus 2020;
- The need for Prednisolone rescue therapy in Cortiment-treated patients;
- The need for hospitalisation;
- Clinical outcomes at the end of treatment.

### 2.2. Statistical Analysis

Descriptive statistics are reported as mean ± SD or proportions. Categorical data were compared between groups using the Chi-square test, whereas the independent samples *t*-test was used for continuous variables. Differences were considered statistically significant if *p* < 0.05. IBM SPSS version 25 (IBM Corp., Armonk, NY, USA) and was used for statistical analysis.

## 3. Results

### 3.1. Comparison of 2019 and 2020 Cohorts

We identified 221 patients, 94 in 2019 and 127 in 2020 (Table 1), that were prescribed steroids, either Cortiment or Prednisolone, for a flare in UC. There was no statistically significant difference between the 2020 group and the 2019 control group with regards to patient age (41.6 vs. 42.06 years, *p* = 0.833), sex (*p* = 0.986), or disease distribution (*p* = 0.136). Moreover, the two groups did not differ significantly on baseline characteristics, including baseline bowel frequency (mean 7.24 vs. 7.64, *p* = 0.519), physician global assessment at baseline (*p* = 0.149), rectal bleeding at baseline (*p* = 0.336), and pMAYO score at baseline (M_2019_ = 6.04 vs. M_2020_ = 6.20, *p* = 0.5). The proportion of patients that were currently on mesalazine was 68.1% in 2019 and 68.5% in 2020 (*p* = 0.947) and similarly there was no statistically significant difference in the number of patients that were currently on thiopurines (23.4% vs. 15.7%, *p* = 0.152) and biologics (21.3% vs. 24.4%, *p* = 0.585) between 2019 and 2020.

The proportion of patients prescribed Prednisolone fell significantly from 75.5% in 2019 to 29.9% in 2020, whereas the proportion of Cortiment prescriptions rose significantly from 24.5% to 70.1% (*p* < 0.001). Statistically significant differences in Cortiment use between 2019 and 2020 were observed among patients that were currently on mesalazine (25% in 2019 vs. 75.9% in 2020, *p* < 0.001) and thiopurines (9.1% in 2019 vs. 65% in 2020, *p* < 0.001), but not for patients on biologics (*p* = 0.137).

The mesalazine dose was altered in 18.5% of patients in 2019 compared to 21.6% in 2020 (*p* = 0.572), whereas there was a statistically significant difference in the alterations in rectal therapy in 2020 compared to 2019 with 41.3% of patients having changes in rectal therapy in 2020 compared to 17.4% in 2019 (*p* < 0.001). There was no significant difference observed in the number of hospital admissions or the number of patients who commenced a new biologic or had their dose altered between 2019 and 2020 (*p* = 0.418 and *p* = 0.949, respectively).

The mean bowel frequency at four weeks was 3.49 in 2019 compared to 5.85 in 2020 (*p* = 0.001) and there was also a statistically significant difference observed in rectal bleeding at week four (89.7% of patients in 2019 reported bleeding in <50% of bowel motions compared to 73.1% in 2020, *p* = 0.039), physician global assessment at week four (39.2% of patients were well in 2019 compared to 19.8% in 2020, *p* = 0.045), as well as pMAYO score at week four of treatment (M = 3.21 in 2019 vs. M = 4.62 in 2020, *p* < 0.001) when comparing the group from 2020 to the historic control group of 2019.

There was a statistically significant difference in the proportion of patients in whom symptoms did not improve within four weeks of therapy (18.1% in 2019 vs. 28.3% in 2020, *p* = 0.039). Moreover, the number of recorded cases that flared after initial improvement differed significantly between 2019 and 2020 (21.6% in 2019 vs. 37.8% in 2020, *p* = 0.031).

Finally, there was no statistically significant difference between 2019 and 2020 on the physician global assessment at the end of treatment (*p* = 0.134), nor on the number of patients on Cortiment that required rescue therapy with Prednisolone at week eight of treatment (6.25% in 2019 vs. 12.5% in 2020, *p* = 0.488).

### 3.2. Comparison of Cortiment Treatment Outcomes in 2019 versus 2020

When comparing patients prescribed Cortiment between 2019 and 2020 (Table 2), there was no statistically significant difference in age (M_2019_ = 40y vs. M_2020_ = 42.33y, *p* = 0.508), sex (*p* = 0.286), current use of mesalazine (69.6% in 2019 vs. 74.2% in 2020, *p* = 0.658), thiopurines (8.7% in 2019 vs. 14.6% in 2020, *p* = 0.458), or biologics (34.8% in 2019 vs. 21.3%, *p* = 0.179). On the other hand, disease extension differed significantly between 2019 and 2020 with 4.4% of patients having proctitis in 2019 compared to 31.5% in 2020 (*p* = 0.021).

Mean bowel frequency at baseline was 6.53 in 2019 not differing significantly from the mean bowel frequency in 2020 which was 7.58 (*p* = 0.335). Moreover, there was no statistically significant difference in rectal bleeding at the baseline (*p* = 0.628), physician global assessment at the baseline (*p* = 0.219), or pMAYO score at the baseline (M = 5.62 in 2019 vs. M = 6.13 in 2020, *p* = 0.223).

The mesalazine dose was altered in 26.1% of patients in 2020 compared to 9.1% in 2019 (*p* = 0.088), whereas a significantly higher proportion of patients had rectal therapy added in 2020 compared to 2019 (51.7% in 2020 vs. 22.7% in 2019, *p* = 0.015). Finally, there was no statistically significant difference between 2019 and 2020 in the proportion of patients started on biologics (*p* = 0.062) or requiring hospital admission for colitis (*p* = 0.139).

The mean bowel frequency at four weeks was significantly higher in 2020 compared to 2019 (M_2019_ = 3.69 vs. M_2020_ = 6.18, *p* = 0.034) and there was also a statistically significant difference in pMAYO score observed (M_2019_ = 3.5 vs. M_2020_ = 4.93, *p* = 0.028), but rectal bleeding at week four and physician global assessment at week four did not differ between 2019 and 2020 (*p* = 0.388 and *p* = 0.422 respectively). Among the patients prescribed Cortiment, 10% of patients in 2019 required rescue Prednisolone at week four of treatment, compared to 31.3% of patients in 2020; however, this difference was not statistically significant (*p* = 0.058).

The proportion of patients on Cortiment that flared after initial improvement differed significantly between 2019 and 2020 (6.7% in 2019 vs. 39.6% in 2020, *p* = 0.017); however, the was no statistically significant difference in rectal bleeding, physician global assessment, or the need for rescue prednisolone at the end of treatment (*p* = 0.092, *p* = 0.180 and *p* = 0.488, respectively).

### 3.3. Comparison of Prednisolone Results in 2019 versus Cortiment Results in 2020

We identified 71 patients who were prescribed prednisolone in 2019 and 89 patients prescribed Cortiment in 2020 (Table 3). There was no statistically significant difference between the two groups with regards to age (M_Pred2019_ = 42.73y vs. M_Corti2020_ = 42.33y, *p* = 0.881), sex (*p* = 0.250), disease distribution (*p* = 0.151), current use of mesalazine (67.6% in Prednisolone group vs. 74.2% in Cortiment group, *p* = 0.363), or biologics (16.9% in Prednisolone group vs. 21.3% in Cortiment group, *p* = 0.480), whereas a significantly higher percentage of patients were currently on thiopurines in the Prednisolone group compared to the Cortiment group (28.2% vs. 14.6%, *p* = 0.035).

Mean bowel frequency at the baseline was 7.46 for the Prednisolone group compared to 7.58 for the Cortiment group (*p* = 0.868); moreover, the two groups did not differ significantly with regards to the pMAYO score (M_Pred2019_ = 6.16 vs. M_Corti2020_ = 6.13, *p* = 0.916), or rectal bleeding at baseline (*p* = 0.079), however, there was a statistically significant difference at the physician global assessment with 13.1% of the Prednisolone group having mild disease compared to 30.8% in the Cortiment group (*p* = 0.036).

A significantly higher proportion of patients in the Cortiment group had rectal therapy added compared to the Prednisolone group (51.7% vs. 15.7%, *p* < 0.001), whereas there was no difference observed between the two groups in the alterations in mesalazine use (*p* = 0.492), in-hospital admissions (*p* = 0.829), or commencement of biologics (*p* = 0.562).

The mean bowel frequency at four weeks of treatment was significantly higher for the group of patients who were prescribed Cortiment in 2020 compared to those prescribed Prednisolone in 2019 (M_Pred2019_ = 3.42 vs. M_Corti2020_ = 6.18, *p* < 0.001), and there was a statistically significant difference observed in the pMAYO score (M_Pred2019_ = 3.11 vs. M_Corti2020_ = 4.93, *p* < 0.001) as well. Moreover, 60.6% of the patients in the Prednisolone group had improvement in their symptoms at week four of treatment compared to 39.3% in the Cortiment group (*p* = 0.014). Finally, no statistically significant differences were observed between the two groups with regards to rectal bleeding and physician global assessment at four weeks of treatment (*p* = 0.11 and *p* = 0.12, respectively) and physician global assessment at end of treatment (*p* = 0.231), whereas 94.2% of the patients in the Prednisolone group had <50% of bleeding at end of treatment compared to 74.5% in the Cortiment group (*p* = 0.015).

## 4. Discussion

The outbreak of the COVID-19 pandemic had a significant impact on the management of patients with IBD worldwide as access to IBD services became more difficult and concerns over immunosuppressive treatments arose. Steroids were seen as a potential risk for IBD patients early in the pandemic [9,12]. Hence, the BSG recommended avoiding systemic corticosteroids if possible and considering Cortiment as the alternative. This change in practice is reflected in the results of this multi-centre study, where the proportion of Cortiment prescriptions rose significantly from 24.5% in 2019 to 70.1% in 2020 (*p* < 0.001). Our study examines the outcomes and consequences of this policy shift.

This study demonstrates the change in the pattern of prescribing steroids in 2020 that resulted in increased use of Cortiment and was associated with worse disease outcomes at four weeks of treatment. The mean bowel frequency at four weeks was significantly higher in 2020 compared to 2019 (*p* = 0.001), there was more rectal bleeding observed (*p* = 0.039), and fewer patients were in remission according to the physician global assessment (*p* = 0.045). Moreover, a significantly higher proportion of patients did not improve within four weeks of treatment (*p* = 0.039) and more cases were recorded in 2020 compared to 2019 that flared after initial improvement (*p* = 0.031). Our findings suggest Cortiment use was associated with poorer disease control at four weeks. This is further underscored by the fact that a greater proportion of patients on Cortiment required rescue therapy with prednisolone by week four (numerical increase from 10% in 2019 to 31.3% in 2020, *p* = 0.058). This may indicate that Cortiment did not provide sufficient efficacy for many patients and the aim of avoiding systemic corticosteroids was not achieved in nearly a third of the 2020 cases.

The registration studies for Cortiment assessed the efficacy for the treatment of mild to moderate UC [5,6]. Yet, in routine clinical practice, Cortiment is often used in patients already established on immunosuppressive therapies and, therefore, by definition, in cases classed as moderate to severe UC. This use of Cortiment in clinical scenarios not examined by randomised controlled trial evidence may explain some of the limited efficacy seen in our study, especially when used as first-line therapy in 2020. We also compared the group of patients who received Prednisolone in 2019 to those treated with Cortiment in 2020. In 2019, 13.1% of the Prednisolone group had mild disease at the baseline compared to 30.8% in the 2020 Cortiment group (*p* = 0.036). This indicates that the Cortiment group of patients would be easier to treat as the proportion of mild disease was higher. Despite this, the mean bowel frequency at four weeks of treatment was significantly higher for the group of patients who were prescribed Cortiment compared to those prescribed Prednisolone (*p* < 0.001). In addition, a significantly higher percentage of the patients in the Prednisolone group had improvement in their symptoms at week four of treatment (*p* = 0.014).

Our data provide evidence that Cortiment is not as efficacious as Prednisolone in real-world clinical practice. Cortiment has proven to be more effective than placeboes in the induction of remission in active mild to moderate UC [5,6]. However, pooled analysis of the CORE I and CORE II studies failed to demonstrate benefit over placeboes in extensive UC [13]. Moreover, there are currently no adequately powered studies comparing the efficacy of Cortiment and conventional corticosteroids. Results from our study indicate that treatment with Cortiment was associated with worse outcomes at four weeks of treatment compared to treatment with Prednisolone. Further studies comparing Cortiment to Prednisolone are needed.

Our study has significant implications on clinical practice during the pandemic. Earlier studies considered active IBD as a significant risk factor for adverse COVID-19 outcomes [12]. While systemic corticosteroids can have a detrimental effect on COVID-19 outcomes we must choose effective treatment to get IBD patients into remission quickly. Therefore, clinicians must weigh the risks of a flare versus the risk of systemic corticosteroids when steroids are required to manage IBD. As highlighted in a RAND process from the British Society of Gastroenterology for acute severe UC, choosing evidence-based strategies remain important through the pandemic [14]. We would, therefore, suggest that patients with moderate to severe UC be treated with systemic corticosteroids, while in those with mild to moderate disease Cortiment may be the most appropriate choice during the pandemic.

There are several limitations to our study. Firstly, this was a retrospective cohort study and not a controlled trial with the obvious lack of controlled circumstances and predefined assessments. Moreover, due to the retrospective nature of the study, there was no evaluation of biochemical markers or endoscopic indices included in the study. Furthermore, data on side effects were not consistently recorded and, therefore, not analysed to avoid bias. The changes in practice occurred at three UK hospitals but this may not necessarily reflect the wider UK or even international practice. We carefully considered whether prescription practice for Cortiment differed from prednisolone. As we did not find any statistically significant differences at the baseline, we are reassured that it was appropriate to compare the cohorts. We did, however, not collect any data on previous flares in the 12 months leading up to the study periods. We did not collect data on the incidence of the SARS-CoV-2 infection or COVID-19 disease for the 2020 cohort. The interesting research question of whether Cortiment vs. prednisolone prescribing influences the likelihood of acquiring COVID-19, unfortunately, cannot be answered with our data set. While it is standard practice to collect stool cultures to examine for superinfection at the time of a flare, we did not collect these data. Finally, a larger sample size might be needed to fully evaluate the possible side effects.

## 5. Conclusions

The outbreak of the COVID-19 pandemic posed drastic changes for the management of IBD, in general, and flares of UC more specifically. This multi-centre study demonstrated that the increased use of Cortiment was associated with worse outcomes at four weeks of treatment and 31% of patients treated with Cortiment needed rescue Prednisolone. Further research with head-to-head comparative trials between Cortiment and conventional steroids is needed. As active IBD is associated with worse COVID-19 outcomes, clinicians should carefully evaluate the choice of steroid to achieve optimal disease control and COVID-19 risk minimization.

## Figures and Tables

**Table 1:** Comparison of 2019 and 2020 cohorts.

		2019 Cohort	2020 Cohort	*p*-Value
Age, Mean (y)		42.06	41.6	0.833
Sex, *n* (%)	Male	48 (51.1)	65 (51.2)	0.986
	Female	46 (48.9)	62 (48.8)	
Disease extent, *n* (%)	E1	15 (16.1)	34 (26.8)	0.136
	E2	46 (49.5)	50 (39.4)	
	E3	32 (34.4)	43 (33.8)	
Bowel Frequency Baseline, Mean		7.24	7.64	0.519
Rectal Bleeding Baseline, *n* (%)	<50%	44 (55.7)	44 (44.9)	0.336
	>50%	26 (32.9)	42 (42.9)	
	Always blood	9 (11.4)	12 (12.2)	
Physician Global Assessment Baseline, *n* (%)	Well	0 (0)	1 (0.9)	0.149
	Mild	15 (18.5)	32 (29.4)	
	Moderate	52 (64.2)	53 (48.6)	
	Severe	14 (17.3)	23 (21.1)	
pMAYO Score Baseline, Mean		6.04	6.20	0.5
Current Mesalazine, *n* (%)	Yes	64 (68.1)	87 (68.5)	0.625
	No	30 (31.9)	40 (31.5)	
Current Thiopurines, *n* (%)	Yes	22 (23.4)	72 (76.6)	0.343
	No	20 (15.7)	107 (84.3)	
Current Biologics, *n* (%)	Yes	20 (21.3)	74 (78.7)	0.585
	No	31 (24.4)	96 (75.6)	
Steroid, *n* (%)	Prednisolone	71 (75.5)	38 (29.9)	0.001
	Cortiment	23 (24.5)	89 (70.1)	
Mesalazine dose alteration, *n* (%)	No	75 (81.5)	98 (78.4)	0.572
	Yes	17 (18.5)	27 (21.6)	
Rectal therapy alteration, *n* (%)	No	76 (82.6)	74 (58.7)	0.001
	Yes	16 (17.4)	52 (41.3)	
Biologic commencement/dose alteration, *n* (%)	No	67 (73.6)	94 (74)	0.949
	Yes	24 (26.4)	33 (26)	
Hospital admission, *n* (%)	No	71 (86.6)	88 (82.2)	0.418
	Yes	11 (13.4)	19 (17.8)	
Bowel Frequency Week 4, Mean		3.49	5.85	0.001
Rectal Bleeding Week 4, *n* (%)	<50%	61 (89.7)	57 (73.1)	0.039
	>50%	4 (5.9)	12 (15.4)	
	Always blood	3 (4.4)	9 (11.5)	
Physician Global Assessment Week 4, *n* (%)	Well	29 (39.2)	18 (19.8)	0.045
	Mild	24 (32.4)	38 (41.7)	
	Moderate	14 (18.9)	20 (22)	
	Severe	7 (9.5)	15 (16.5)	
pMAYO Score Week 4, Mean		3.21	4.62	0.001
Symptom improvement within 4 weeks, *n* (%)	No	17 (18.1)	36 (28.3)	0.039
	Yes	56 (59.6)	54 (42.5)	
Flare after initial improvement, *n* (%)	No	58 (78.4)	46 (62.2)	0.031
	Yes	16 (21.6)	28 (37.8)	
Physician Global Assessment Week 8, *n* (%)	Well	30 (41.7)	20 (25.6)	0.134
	Mild	21 (29.2)	28 (35.9)	
	Moderate	17 (23.6)	20 (25.6)	
	Severe	4 (5.5)	10 (12.8)	
Rescue Prednisolone Week 4, *n* (%)	No	18 (85.7)	46 (68.7)	0.126
	Yes	3 (14.3)	21 (31.3)	
Rescue Prednisolone Week 8, *n* (%)	No	15 (93.75)	42 (87.5)	0.488
	Yes	1 (6.25)	6 (12.5)	

**Table 2:** Comparison of Cortiment treatment outcomes in 2019 versus 2020.

		Cortiment 2019	Cortiment 2020	*p*-Value
Age, Mean (y)		40	42.33	0.508
Sex, *n* (%)	Male	8 (34.8)	42 (47.2)	0.286
	Female	15 (65.2)	47 (52.8)	
Disease extent, *n* (%)	E1	1 (4.4)	28 (31.5)	0.021
	E2	13 (56.5)	30 (33.7)	
	E3	9 (39.1)	31 (34.8)	
Bowel Frequency Baseline, Mean		6.53	7.58	0.335
Rectal Bleeding Baseline, *n* (%)	<50%	10 (55.5)	30 (42.9)	0.628
	>50%	7 (38.9)	35 (50)	
	Always blood	1 (5.6)	5 (7.1)	
Physician Global Assessment Baseline, *n* (%)	Well	0 (0)	0 (0)	0.219
	Mild	7 (35)	24 (30.8)	
	Moderate	12 (60)	37 (47.4)	
	Severe	1 (5)	17 (21.8)	
pMAYO Score Baseline, Mean		5.62	6.13	0.223
Current Mesalazine, *n* (%)	Yes	16 (69.6)	66 (74.2)	0.658
	No	7 (30.4)	23 (25.8)	
Current Thiopurines, *n* (%)	Yes	2 (8.7)	13 (14.6)	0.458
	No	21 (91.3)	76 (85.4)	
Current Biologics, *n* (%)	Yes	8 (34.8)	19 (21.3)	0.179
	No	15 (65.2)	70 (78.7)	
Mesalazine dose alteration, *n* (%)	No	20 (90.9)	65 (73.9)	0.088
	Yes	2 (9.1)	23 (26.1)	
Rectal therapy alteration, *n* (%)	No	17 (77.3)	43 (48.3)	0.015
	Yes	5 (22.7)	46 (51.7)	
Biologic commencement/dose alteration, *n* (%)	No	13 (61.9)	72 (80.9)	0.062
	Yes	8 (38.1)	17 (19.1)	
Hospital admission, *n* (%)	No	20 (95.2)	60 (82.2)	0.139
	Yes	1 (4.8)	13 (17.8)	
Bowel Frequency Week 4, Mean		3.69	6.18	0.034
Rectal Bleeding Week 4, *n* (%)	<50%	16 (84.2)	36 (67.9)	0.388
	>50%	2 (10.5)	10 (18.9)	
	Always blood	1 (5.3)	7 (13.2)	
Physician Global Assessment Week 4, *n* (%)	Well	5 (26.3)	10 (16.1)	0.422
	Mild	8 (42.1)	27 (43.5)	
	Moderate	5 (26.3)	13 (21)	
	Severe	1 (5.3)	12 (19.4)	
pMAYO Score Week 4, Mean		3.5	4.93	0.028
Symptom improvement within 4 weeks, *n* (%)	No	6 (26.1)	29 (32.6)	0.314
	Yes	13 (56.5)	35 (39.3)	
Flare after initial improvement, *n* (%)	No	14 (93.3)	29 (60.4)	0.017
	Yes	1 (6.7)	19 (39.6)	
Physician Global Assessment Week 8, *n* (%)	Well	8 (47.1)	12 (23.1)	0.180
	Mild	6 (35.3)	21 (40.4)	
	Moderate	3 (17.6)	13 (25)	
	Severe	0 (0)	6 (11.5)	
Rescue Prednisolone Week 4, *n* (%)	No	18 (90)	46 (68.7)	0.058
	Yes	2 (10)	21 (31.3)	
Rescue Prednisolone Week 8, *n* (%)	No	15 (93.7)	42 (87.5)	0.488
	Yes	1 (6.3)	6 (12.5)	

**Table 3:** Comparison of Prednisolone results in 2019 versus Cortiment results in 2020.

		Prednisolone 2019	Cortiment 2020	*p*-Value
Age, Mean (y)		42.73	42.33	0.881
Sex, *n* (%)	Male	40 (56.3)	42 (47.2)	0.250
	Female	31 (43.7)	47 (52.8)	
Disease extent, *n* (%)	E1	14 (20)	28 (31.5)	0.151
	E2	33 (47.1)	30 (33.7)	
	E3	23 (32.9)	31 (34.8)	
Bowel Frequency Baseline, Mean		7.46	7.58	0.868
Rectal Bleeding Baseline, *n* (%)	<50%	34 (55.7)	30 (42.9)	0.079
	>50%	19 (31.1)	35 (50)	
	Always blood	8 (13.1)	5 (7.1)	
Physician Global Assessment Baseline, *n* (%)	Well	0 (0)	0 (0)	0.036
	Mild	8 (13.1)	24 (30.8)	
	Moderate	40 (65.6)	37 (47.4)	
	Severe	13 (21.3)	17 (21.8)	
pMAYO Score Baseline, Mean		6.16	6.13	0.916
Current Mesalazine, *n* (%)	Yes	48 (67.6)	66 (74.2)	0.363
	No	23 (32.4)	23 (25.8)	
Current Thiopurines, *n* (%)	Yes	20 (28.2)	13 (14.6)	0.035
	No	51 (71.8)	76 (85.4)	
Current Biologics, *n* (%)	Yes	12 (16.9)	19 (21.3)	0.480
	No	59 (83.1)	70 (78.7)	
Mesalazine dose alteration, *n* (%)	No	55 (78.6)	65 (73.9)	0.492
	Yes	15 (21.4)	23 (26.1)	
Rectal therapy alteration, *n* (%)	No	59 (84.3)	43 (48.3)	0.001
	Yes	11 (15.7)	46 (51.7)	
Biologic commencement/dose alteration, *n* (%)	No	54 (77.1)	72 (80.9)	0.562
	Yes	16 (22.9)	17 (19.1)	
Hospital admission, *n* (%)	No	51 (83.6)	60 (82.2)	0.829
	Yes	10 (16.4)	13 (17.8)	
Bowel Frequency Week 4, Mean		3.42	6.18	0.001
Rectal Bleeding Week 4, *n* (%)	<50%	45 (91.8)	36 (67.9)	0.11
	>50%	2 (4.1)	10 (18.9)	
	Always blood	2 (4.1)	7 (13.2)	
Physician Global Assessment Week 4, *n* (%)	Well	24 (43.6)	10 (16.1)	0.12
	Mild	16 (29.1)	27 (43.5)	
	Moderate	9 (16.4)	13 (21)	
	Severe	6 (10.9)	12 (19.4)	
pMAYO Score Week 4, Mean		3.11	4.93	0.001
Symptom improvement within 4 weeks, *n* (%)	No	11 (15.5)	29 (32.6)	0.014
	Yes	43 (60.6)	35 (39.3)	
Flare after initial improvement, *n* (%)	No	44 (74.6)	29 (60.4)	0.118
	Yes	15 (25.4)	19 (39.6)	
Physician Global Assessment Week 8, *n* (%)	Well	22 (40)	12 (23.1)	0.231
	Mild	15 (27.3)	21 (40.4)	
	Moderate	14 (25.4)	13 (25)	
	Severe	4 (7.3)	6 (11.5)	

## Data Availability

As there is no patient consent for data sharing all study data are not available publicly.
